# Supplementary material for: GplR1, an unusual TetR-like transcription factor in Mycobacterium abscessus, controls the production of cell wall glycopeptidolipids, colony morphology, and virulence
Source: mSystems. 2025 Aug 28;10(9):e00872-25. doi: 10.1128/msystems.00872-25 (PMC12455991; doi:10.1128/msystems.00872-25)
Supplement: Supplemental Figures — Fig. S1 to S3. [file msystems.00872-25-s0001.pdf]

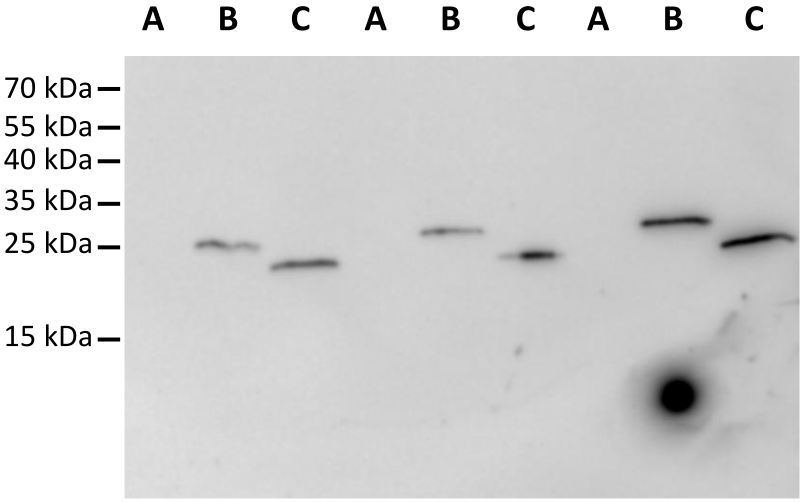

A: WT (no HA-tagged protein)

B: *mab_1638*::tn + *mab_1638-HA* (smooth)

C: *mab_1638*::tn + *mab_1638*$^{\Delta AA43\text{-}62}$*-HA* (rough)

Supplementary Figure 1: Deletion of the DNA binding region of MAB_1638 does not lead to instability of the protein. The *mab_1638*::tn mutant was complemented with an HA-tagged version of full-length *mab_1638* gene or an HA-tagged variant with a deletion of the predicted DNA binding region (*mab_1638*$^{\Delta AA43\text{-}62}$). The expected molecular weights of the two tagged variants of MAB_1638 are 24.6 kDa and 22.2 kDa. Triplicate cultures of the two complemented strains and WT *M. abscessus* ATCC19977 were grown to log phase, lysates were separated on a polyacrylamide gel and subjected to western blotting against HA. The data are representative of two independent experiments.

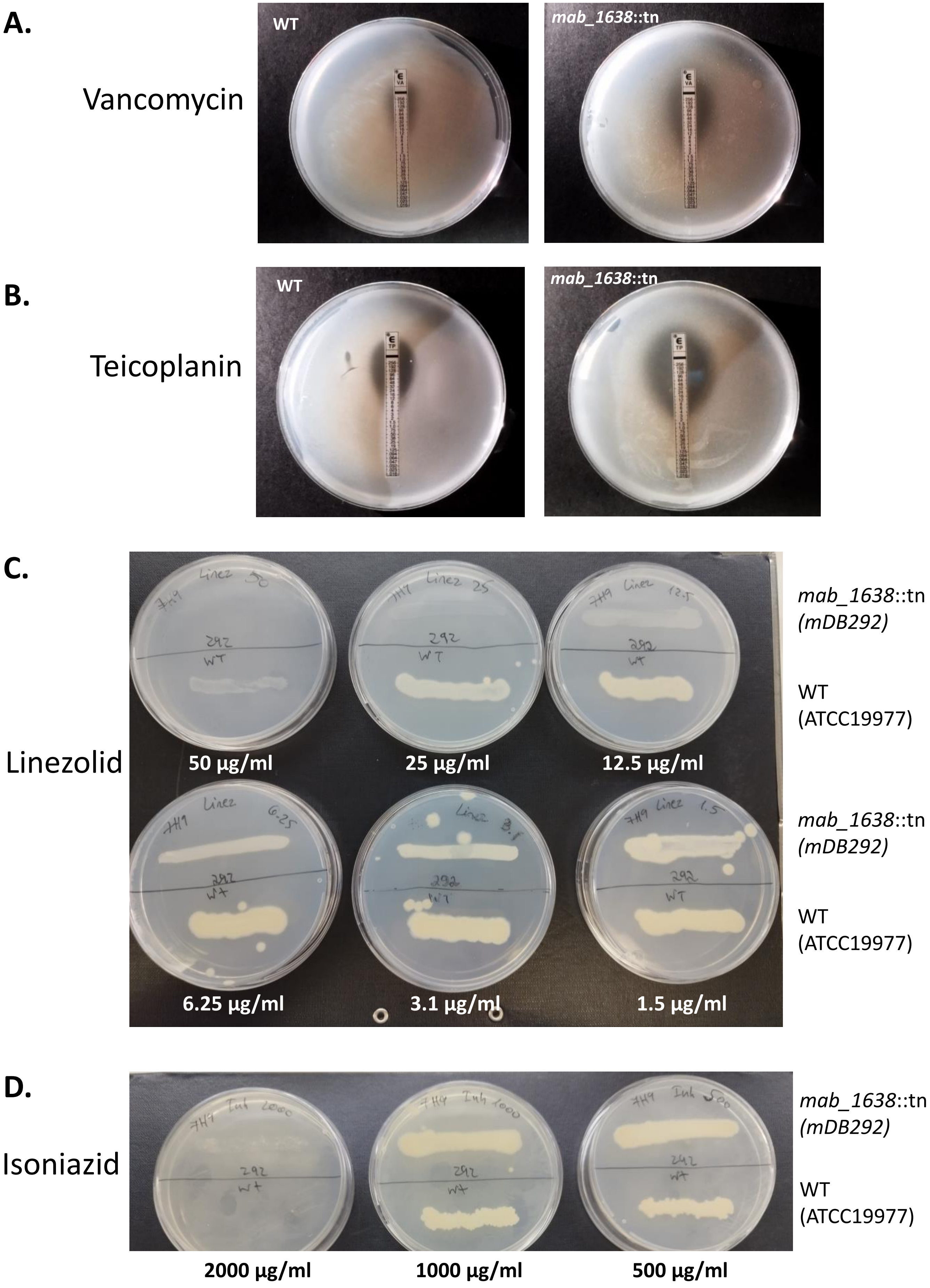
A.
Vancomycin
WT
mab_1638::tn
B.
Teicoplanin
WT
mab_1638::tn
C.
Linezolid
50 µg/ml
25 µg/ml
12.5 µg/ml
6.25 µg/ml
3.1 µg/ml
1.5 µg/ml
mab_1638::tn *(mDB292)*
WT (ATCC19977)
mab_1638::tn *(mDB292)*
WT (ATCC19977)
D.
Isoniazid
2000 µg/ml
1000 µg/ml
500 µg/ml
mab_1638::tn *(mDB292)*
WT (ATCC19977)

Supplementary Figure 2: **The inactivation of *mab_1638* in *M. abscessus* causes small increases in sensitivity to some antibiotics.** *Mycobacterium abscessus* WT (ATCC19977) and the *mab_1638*::tn mutant (mDB292) were tested by E-test for the MIC to vancomycin (A), teicoplanin (B), and by agar dilution to linezolid (C) and isoniazid (D). In (C,D), bacteria were streaked on 7H9/glycerol agar plates with the designated concentrations of linezolid (1.5-50 µg/ml) or isoniazid (500-2000 µg/ml).

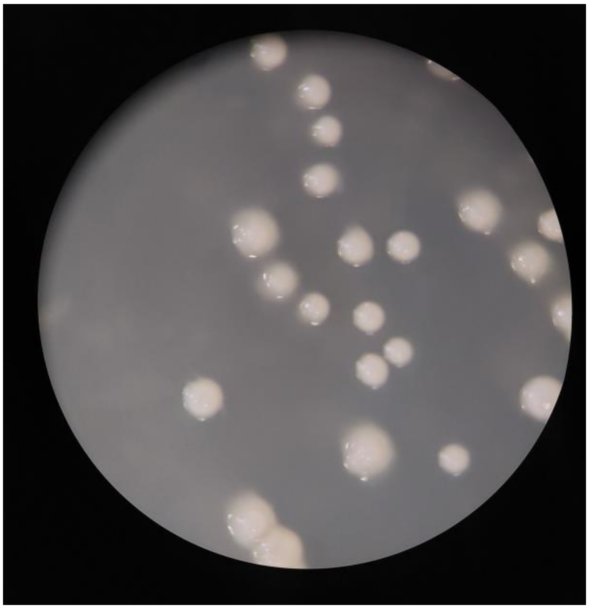

mDB352 (*mab_1638*::tn mutant + *mab_1638-HA tag*. The tag is located at the C-terminus of the protein.

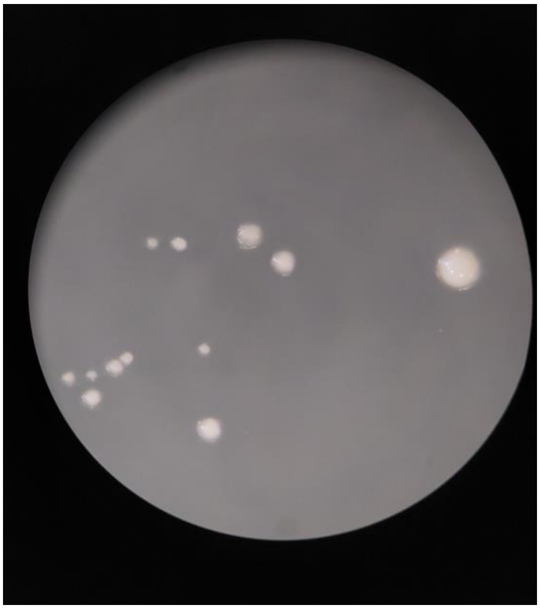

mDB353 (*mab_1638*::tn mutant + *HA tag-mab_1638*. The tag is located at the N-terminus of the protein.

Supplementary Figure 3: HA-tagging of MAB_1638 does not disrupt its function. For the ChiP-seq, the *mab_1638*::tn mutant (rough) was complemented with tagged version of the native *mab_1638* gene, with an HA-tag attached either at the N or the C terminus of the protein. To demonstrate the tagged protein is functional, single colonies were examined for morphology. As seen, both tagged versions of *mab_1638* revert the colony morphology back to smooth, confirming proper function.
